# Genome-wide identification, structural homology analysis, and evolutionary diversification of the phospholipase D gene family in the venom gland of three scorpion species

**DOI:** 10.1186/s12864-023-09851-y

**Published:** 2023-12-04

**Authors:** Masoumeh Baradaran, Fatemeh Salabi

**Affiliations:** 1 Toxicology Research Center, Medical Basic Sciences Research Institute, Ahvaz Jundishapur University of Medical Sciences, Ahvaz, Iran; 2 Department of Venomous Animals and Anti-Venom Production, Agricultural Research, Education and Extension Organization (AREEO), Razi Vaccine and Serum Research Institute, Ahvaz, Iran

**Keywords:** Phospholipase D, Dermonecrotic toxins, Transcriptomic analysis, Conserved regions

## Abstract

**Background:**

Venom phospholipase D (PLDs), dermonecrotic toxins like, are the major molecules in the crude venom of scorpions, which are mainly responsible for lethality and dermonecrotic lesions during scorpion envenoming. The purpose of this study was fivefold: First, to identify transcripts coding for venom PLDs by transcriptomic analysis of the venom glands from *Androctonus crassicauda, Hottentotta saulcyi,* and *Hemiscorpius lepturus*; second, to classify them by sequence similarity to known PLDs and motif extraction method; third, to characterize scorpion PLDs; fourth to structural homology analysis with known dermonecrotic toxins; and fifth to investigate phylogenetic relationships of the PLD proteins.

**Results:**

We found that the venom gland of scorpions encodes two PLD isoforms: PLD1 ScoTox-beta and PLD2 ScoTox-alpha I. Two highly conserved regions shared by all PLD1s beta are GAN and HPCDC (HX2PCDC), and the most important conserved regions shared by all PLD2s alpha are two copies of the HKDG (HxKx4Dx6G) motif. We found that PLD1 beta is a 31–43 kDa acidic protein containing signal sequences, and PLD2 alpha is a 128 kDa basic protein without known signal sequences. The gene structures of PLD1 beta and PLD2 alpha contain 6 and 21 exons, respectively. Significant structural homology and similarities were found between the modeled PLD1 ScoTox-beta and the crystal structure of dermonecrotic toxins from *Loxosceles intermedia*.

**Conclusions:**

This is the first report on identifying PLDs from *A. crassicauda and H. saulcyi* venom glands. Our work provides valuable insights into the diversity of scorpion PLD genes and could be helpful in future studies on recombinant antivenoms production.

**Supplementary Information:**

The online version contains supplementary material available at 10.1186/s12864-023-09851-y.

## Background

Envenoming caused by scorpion species, including *Androctonus crassicauda, Hottentotta saulcyi, Hottentotta schach, Mesobuthus eupeus, Odontobuthus doriae,* and *Hemiscorpius lepturus,* are public health challenges in Iran [1], among which *H. lepturus*, *H. saulcyi*, and *A. crassicauda* sp., are considered as the deadliest scorpions of Iran. Therefore, studying their venom is of particular importance. The spatial distribution of these important medical scorpions in Iran has already been reported [2]. In Brief, *H. lepturus* is regarded as the first deadly scorpion that is distributed in the south and southwest of Iran. The sting of this scorpion is mostly cytotoxic. The second deadly scorpion of Iran, *A. crassicauda* scorpion, is widely distributed and found in the majority of Iran's provinces. The range of this scorpion extends from southern Zagros Mountain to the coastline of the Persian Gulf and Oman Sea. In Iran, *H. saulcyi* scorpions also inhabit in several provinces, primarily from the northwest to the southwest of the country. The *A. crassicauda* and *H. saulcyi* have a neurotoxic venom with an excruciating sting that causes cardiovascular problems.

Each year, a variety of symptoms, including local pain, sweating, vomiting, delirium, anxiety, necrosis, inflammation, muscle paralysis, even hematuria, and extended edema following scorpion sting, are announced by the Iranian Ministry of Health [3, 4]. Many studies have been conducted to find the major contributing factor that causes these symptoms, most of which are based on identifying venom compounds.

Venom of scorpions contains complex mixtures of different biologically active compounds, including enzymes (such as hyaluronidases, phospholipases, and proteases), toxic peptides, free amino acids, carbohydrates, lipids, and other metabolites [5, 6]. Some of the venom components are critical for the scorpion's life because the scorpion uses them for capturing prey, defending against predators, and reproduction [5]. However, many studies investigated that some peptides and proteins of scorpion venoms have cytotoxic, neurotoxic, and immunosuppressive properties [7]. Among the complex secretory mixtures of venom, phospholipases D (PLDs) were identified as the most abundant enzymes with many isoforms. These hydrolase enzymes are the main enzymes of venom that are known to be responsible for the symptoms of neurotoxicity and lethality during envenomation and may be involved in the adaptation of the organism, and the effectiveness of the venom [8]. Phospholipase D (PLD) is known to be a ubiquitous enzyme; its activity has been discovered in a range of species, such as plants, mammals, and microorganisms [9]. In the venom of the *Loxosceles* spider, the phospholipase D (PLD) can transform sphingomyelin (SM) to ceramide 1-phosphate (C1P) by degradation of cell membrane phospholipids [10]. Subsequently, the release of ceramide 1-phosphate (C1P) can proceed to a pro-inflammatory mediator, which can regulate the TNF-α and recruit neutrophils [10, 11]. This enzyme is mainly responsible for dermonecrotic lesions, and these reactions are involved in components of the complement activation, migration of polymorphonuclear leukocytes, platelets aggregation, and inflammatory response [12]. Due to these biochemical characteristics of PLD enzymes of *Loxosceles* venom, the names sphingomyelinase D (SMase D) or dermoncrotic toxin have been widely used by toxicologists.

Furthermore, PLD can associate with a large number of proteins involved in a variety of physiological cellular purposes, such as cytoskeletal dynamics, chemotaxis of leukocytes, and cell proliferation [13, 14]. Although PLD is the most studied and well-known toxin derived from arthropod venoms, especially in the venoms of brown spiders, *Loxosceles* [15], and scorpion venoms of *H. lepturus*, *Centruroides sculpturatus*, *Megacormus gertschi*, and *Paravaejovis schwenkmeyeri* [7, 16–19], there are only general and preliminary studies on scorpion venom PLDs. Therefore, molecular and structural characterization of this enzyme in scorpions is required. In the present study, along with the identification of PLD sequences from venom glands of *A. crassicauda, H. saulcyi,* and *H. lepturus* using de novo transcriptome reconstruction, the classification and characteristics of this enzyme were performed. Furthermore, the current study attempted to predict the exon–intron pattern of the PLD gene of the scorpion venom gland. In addition, the evolutionary relationships between scorpion species and spiders have been developed. This is the first report of the presence of dermonecrotic toxin-like sequences in the venom of *A. crassicauda and H. saulcyi* using a bioinformatics assay.

## Methods

**Fig. 1:**
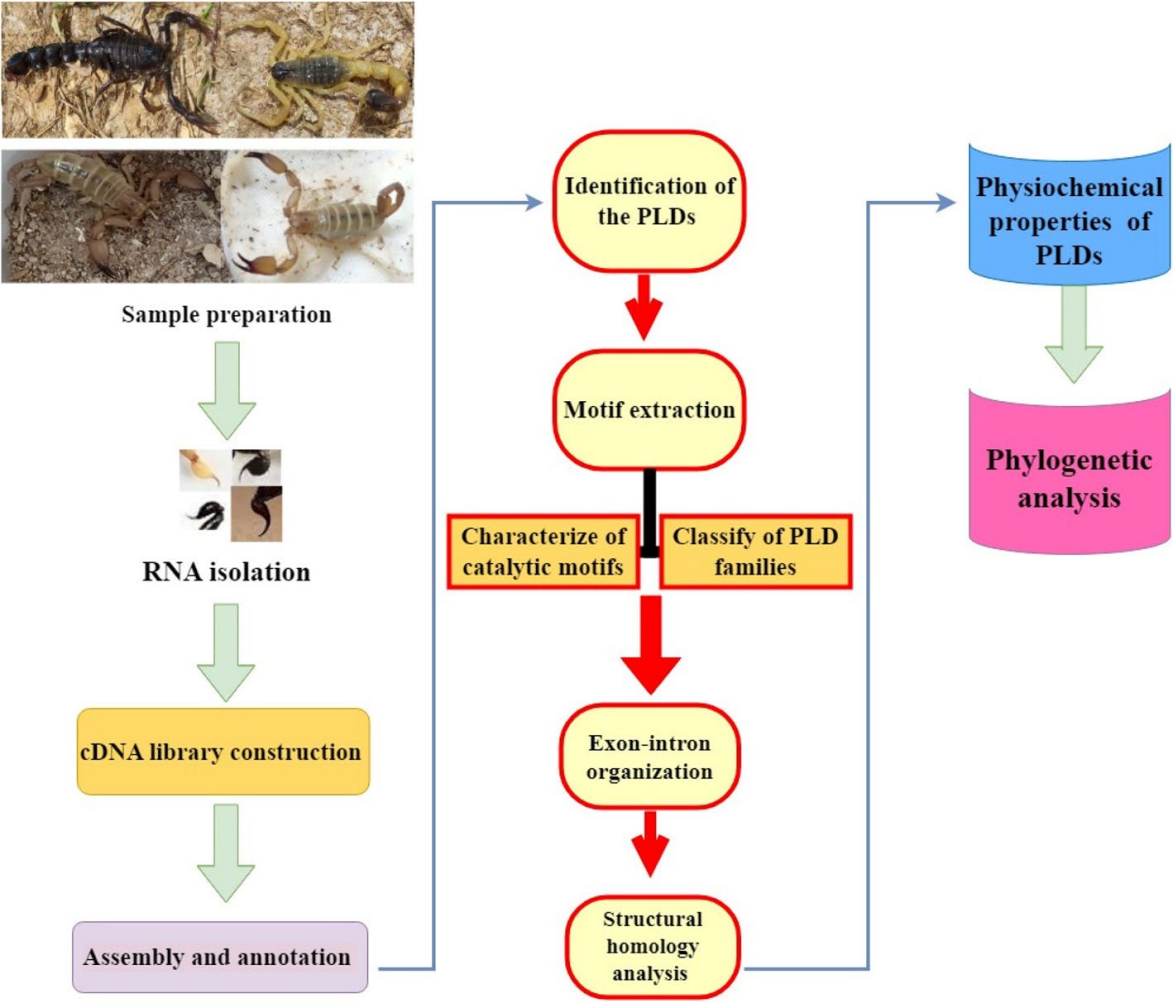
Schematic workflow of the study. This workflow shows the transcriptome analysis of the venom gland of *H. saulcyi*, *H. lepturus*, and *A. crassicauda* scorpions to extract, categorize, and investigate the physicochemical properties and prediction of gene structures of PLDs

This paper presents a transcriptome analysis of the *H. saulcyi* venom gland. *H. lepturus* and *A. crassicauda* cDNA libraries used in this study were produced in our previous study [20]. Detailed information about the experimental design, sample preparation, RNA extraction, and high-throughput RNA-sequencing methods have been described previously [20]. Subsequently, PLDs from scorpions of *H. lepturus*, *A. crassicauda*, and *H. saulcyi* were identified, classified, and characterized. A graphical description of the study steps is given in a workflow diagram (Fig. 1).

### Sample preparation

The *H. saulcyi* scorpions were captured from deserts around the Khuzestan province, south-west of Iran, in autumn 2021 according to ethical guidelines of the World Health Organization (WHO) method. The scorpion species were identified in the laboratory of Razi Vaccine and Serum Research Institute and Toxicology Research Center laboratory of Ahvaz Jundishapur University of Medical Sciences. The milking was carried out by electroshock to allow the venom cells to enter the secretory phase. Three days after milking, the telsons of scorpion individuals were removed and preserved in RNAprotect Tissue Reagent® (Cat. No. /ID: 76,104), for immediate stabilization of RNA.

### RNA isolation and cDNA library construction from venom gland

Total RNA was isolated from freshly isolated venom gland tissues according to the protocol instruction of RNeasy Mini Kit (Cat. 74,104). The quality of extracted RNA was evaluated using Nanodrop (co. Thermo, USA). Each three RNA samples of *H. saulcyi* venom glands were pooled together in equal concentrations to generate two pooled RNA samples. The RNA Integrity Number (RIN) of samples was determined by Macrogen, Inc. (Seoul, Korea) using the Agilent 2100 Bioanalyzer System (Agilent Technologies, USA) according to the manufacturer's instructions. The RNA samples with a RIN > 7 were selected for cDNA library construction. The library was synthesized using TruSeq® Stranded mRNA Library Prep (cat. number 20020594) according to the manufacturer’s instructions. Finally, the cDNA library (from 2 *H. saulcyi* individuals) was sequenced by High-throughput RNA-sequencing (Illumina Next-Generation Sequencing). The samples were subjected to a paired-end sequencing of 150 bp on the Illumina HiSeq 2000 platform (Illumina, Macrogen Co Macrogen, Seoul, Korea).

### Assembly and annotation of deep sequencing data

Trimmomatic and FastQC java programs (http://www.bioinformatics.bbsrc.ac.uk/projects/fastqc/) were used to filter poor quality reads and assess the quality of raw reads, respectively. The clean reads of each individual species were separately de novo assembled into contigs using Trinity software (v2.10.0) [21] with the default parameters. Since no reference sequence is provided for studied scorpion species, the de novo assembled transcriptome sequences were considered reference sequences to evaluate the quality of the transcripts. Bowtie2 v2.3.5.1 was used to map the reads back to the transcriptome assemblies.

Open Reading Frames (ORFs) of the non-redundant transcript isoforms and potential coding sequences were predicted by TransDecoder software v3.0.1 with the ‘single best ORF’ option [22].

### Identification of the PLDs in venom glands transcriptome

To identify the protein and nucleotide sequences with the highest sequence similarities to PLDs and dermonecrotic toxins, the local database was generated using known PLD and dermonecrotic proteins from scorpions and its phylogenetically closely related species from Phylum of Arthropoda, including spider, tick, mite, termite, ant, fly, and wasp. The sequences used to generate the PLDs local database are listed in Additional file 1. Then, the Transdecoder-predicted proteins and nucleotide sequences from each species were searched against the local PLD database using BLASTx and BLASTp with an E value threshold le^−3^. Finally, the homologous sequences representing PLDs were preserved from three scorpion species mRNA candidates. The classification of those sequences was first performed based on sequence similarity to already known families/classes. To classify based on sequence similarity, newly extracted sequences were searched against NCBI and UniProt databases. A sequence alignment was generated with an online server in EBI (https://www.ebi.ac.uk/Tools/msa/clustalo/). For confirmation, we used known motif extraction method by searching the protein sequences against NCBI Conserved Domain Database (CDD) (https://www.genome.jp/tools/motif/), PROSITE collection of motifs online tool (https://prosite.expasy.org/scanprosite/) and RCSB Protein Data Bank (RCSB PDB) (https://www.rcsb.org/search/advanced/sequence). To assist in fast identifying PLD family members from arthropods, including insects and arachnids, we attempted to find a diagnostic peptide pattern by aligning the motif sequences of PLDs from scorpions, spiders, ticks, termites, ants, flies, and wasp species using the MUSCLE tool, a multiple sequence alignment program.

To achieve this purpose, two more scorpion transcriptomes from *Androctonus australis* (SRR1724216.1) and *Hottentotta trilineatus* (SRR1721800.1) were downloaded from NCBI. We conducted de novo assemblies of these transcriptomes with Trinity v2.10.0 [21, 22] and open reading frames prediction with the TransDecoder pipeline (https://transdecoder.github.io/) as described above.

### Characterization of gene and protein of PLDs

To enhance the confidence of the predicted PLDs, the newly discovered protein and nucleotide sequences were subjected to further characterization. An intron–exon map of PLD genes was predicted based on our previous proposed methodology [20], comparing whole genome shotgun sequences with mRNAs. The mRNA sequence of phospholipase D SpeSicTox-beta (XM_023367013.1) with its genomic DNA (NW_019384571.1) and mRNA sequence of phospholipase D2 (XM_023381888.1) with its genomic DNA (NW_019385241.1) from *C. sculpturatus* were used as templates to compare with the PLD mRNA sequences found in this study. A sequence alignment was generated with MAFT (High speed multiple sequence alignments program) online server in EBI (https://mafft.cbrc.jp/alignment/server/index.html).

In the following, the physicochemical properties of the toxin were determined with the ProtParam tool from Expasy (https://web.expasy.org/protparam/) and the proteomics tool from INNOVAGEN (http://pepcalc.com). Molecular weight, iso-electric point, instability index, and water solubility of PLDs were estimated. Potential signal peptides of toxins were predicted via the SignalP-6.0 server (https://services.healthtech.dtu.dk/service.php?SignalP).

### Structural homology analysis

Homology modeling is used to determine the 3-D structures of proteins by in silico prediction methods. By homology modeling, we seek to predict the 3D structures of newly found PLD proteins, and our primary goal is structural alignment between different PLD isoforms, as well as superposition of the predicted structures on the crystal structures of sphingomyelinase D or dermonecrotic toxin from *Loxosceles laeta*. Four models were generated for each protein using four different methods as follows: I-TASSER by multiple threading approaches, Phyre2 with Poing^2^ method, and SWISS-MODEL. The best model was selected via quality assessment using SAVESv6.0 (https://saves.mbi.ucla.edu/) as well as by Z-score through the ProSA-web (https://prosa.services.came.sbg.ac.at/prosa.php). For further analysis of the predicted models, the Ramachandran plot was also analyzed for each structure.

The best quality protein structures were used to compare the structural homology between PLDS from all scorpion species. Visualization and structural alignment of the selected structures of PLDs were performed using UCSF Chimera software (https://www.cgl.ucsf.edu/chimera/). To investigate conformational similarities between multiple structures using UCSF Chimera, the superimposition of the structures was performed using the MatchMaker function with default settings, which is commonly quantified using the root mean squared deviation (RMSD) of the atomic coordinates. In MatchMaker, models with lower RMSD have a higher score of 3D similarity [23].

### Phylogenetic analysis

The homologous peptides of PLDs were extracted using BLASTp by searching for newly found PLDs against the NCBI database. The amino acid sequences of PLDs were then aligned with other representative sequences from scorpions, spiders, ticks, termites, ants, flies, and wasp species using multiple alignments with the MUSCLE tool [24]. The obtained multiple alignment results were used to estimate consensus phylogenetic trees by a Maximum Likelihood Algorithm [25] using the PhyML 3.0 program [26] hosted on the Phylogeny.fr web server (http://www.phylogeny.fr/simple_phylogeny.cgi).

### Ethical statement

The manuscript and data were not previously or simultaneously submitted elsewhere. All experiments in this paper were carried out under the standard procedures of scientific ethics, including the care of experimental animals. Permission was obtained from the Environmental Protection Agency of Iran to collect scorpions of *A. crassicauda*, *H. saulcyi*, and *H. lepturus*. No animals were euthanized as part of this study, and all sample collection methods and experimental procedures described herein were rigorously reviewed and approved by the Institutional Animal Care Committee of Razi Vaccine and Serum Research Institute (Permit number IR.RVSRI.REC.1401.017) and AREEO protocols, which comply with Iran guidelines for work with animals. This study also adheres to the ARRIVE Guidelines for reporting animal research. All procedures were performed following the relevant guidelines in the manuscript. All authors have read the manuscript and agree to its publication in the Journal of Scientific Report and agree that it has followed the rules of ethics presented in the guidelines for journal publication.

## Results

### Assembly and annotation of raw sequences from RNA sequencing

In this work, we carried out a transcriptome analysis of the *H. saulcyi* venom gland for extracting and characterizing the sequences of the phospholipase D. In addition, we used the previously reconstructed venom gland transcriptome from *H. lepturus* and *A. crassicauda* [20]. After sequencing and filtering out low-quality reads, the cDNA library was constructed from approximately 48 million paired ends of clean reads of > 200 bp by the Trinity program [21]. Among the 191,150 assembled transcripts, 110,126 unigenes were obtained, and 98,365 potential coding sequences were predicted using TransDecoder software. We found that a high percentage of those protein-coding sequences (95,167) had a match in non-redundant protein (Nr), Swissprot, and Pfam databases.

In our previous study, following removal of the adaptor, short reads, and quality filtering, approximately 51 and 48 million paired ends of clean reads were obtained in *A. crassicauda* and *H. lepturus*, respectively. In total, 260,663 and 222,989 transcript sequences were reconstructed by de novo transcriptome assembler of Trinity software, in which 152,704 and 122,618 unigenes (> 200 bp) were clustered from deep RNA-Seq data of *A. crassicauda* and *H. lepturus*, respectively. After comparing the protein-coding sequences against NCBI, Swissprot, and Pfam databases, 11,415 and 11,869 transcripts were identified as known mRNAs in *A. crassicauda* and *H. lepturus* transcriptomes, respectively.

### Identification of the scorpion venom PLDs

The first step is building a local database of PLDs to target BLAST searches for phospholipase sequences. This database was created by collecting known PLD sequences from scorpion species and closely related species such as spider, tick, mite, termite, ant, fly, and wasp species listed in Additional file 1. Using our local database, we conducted exhaustive BLAST searches of the *H. saulcyi*, *A. crassicauda*, and *H. lepturus* venom glands transcriptome. We found multiple transcripts with high sequence similarity to PLDs. Generating a target database of PLD sequences from a few widely used databases facilitated the use of comprehensive search strategies to extract subsets of PLD sequences from our dataset. To increase the sequence identification confidence and to classify the sequences, the obtained sequences were directly searched against NCBI and UniProt databases. Sequences sharing high sequence identity with previously classified PLD sequences belonging to the species mentioned above were considered members of this group and subjected to further analysis for classification.

### Domain and motif identification

**Fig. 2:**
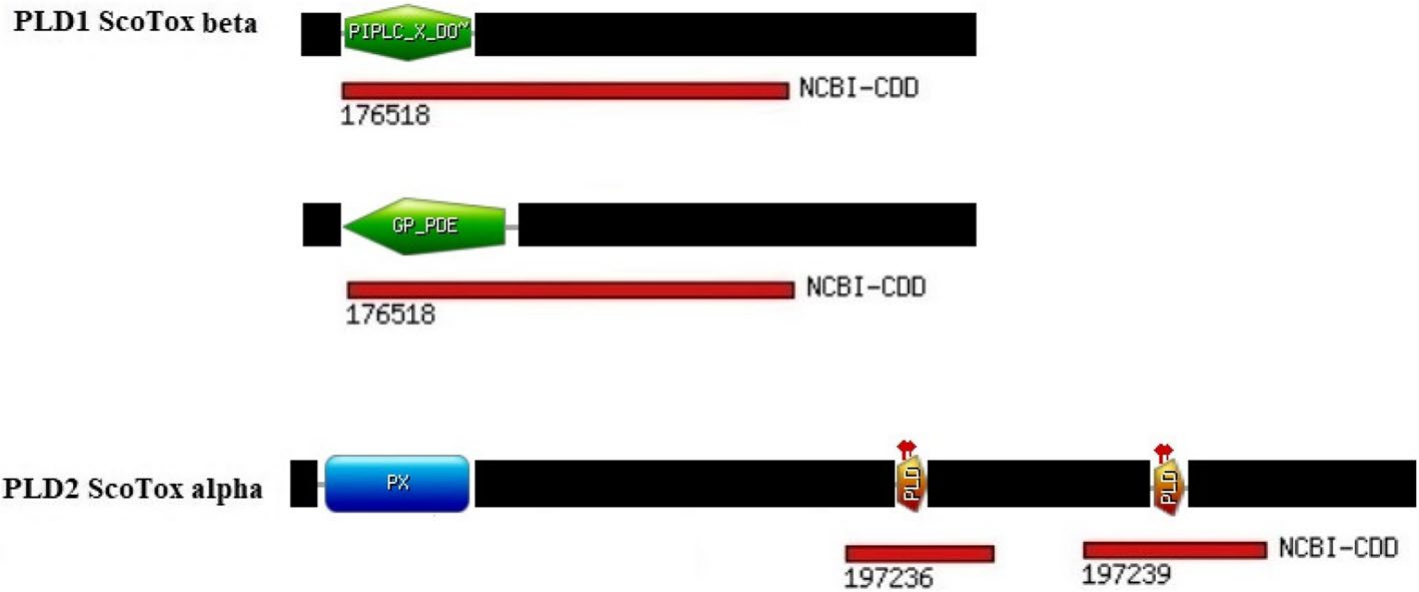
Schematic diagram of conserved motifs and domains in the scorpion PLDs family. Sequence motif searches against the NCBI-CDD and PROSITE databases. In NCBI-CDD database, 176518 shows the Glycerophosphodiester phosphodiesterase-like domain of spider venom sphingomyelinases D (GDPD_like_SMaseD_PLD), 197236 and 197239 are known as the catalytic domain of phospholipases. In the PROSITE database, the domain of PI-PLC shows the phosphatidylinositol-specific phospholipase C, the GP-PDE domain is the glycerophosphodiester phosphodiesterases, the PX domain indicates the phox homology (PX) domain, and the PLD is the phospholipase D phosphodiesterase active site

For definitive categorization, newly obtained PLD sequences were subjected to protein motif and domain identification. For domain identification, we used BLASTp searches of the local copy of the NCBI CDD. Furthermore, the PROSITE collection of motifs online tool and RCSB PDB server were utilized for protein motif exploration. By searching the PROSITE database, we found that some PLD sequences share a motif of phosphatidylinositol-specific phospholipase C (PIPLC_X_DOMAIN) and glycerophosphodiester phosphodiesterases domain (GP-PDE), which were classified as PLD1 beta family. The rest, which share similar motifs, including two copies of HKD motif (PLD) and a phox homology domain (PX), were considered as PLD2 alpha family. By analyzing the results of searches for protein motifs in the NCBI CDD database, two copies of catalytic domains of phospholipases were found for sequences classified into the PLD2 alpha family. In contrast, a Glycerophosphodiester phosphodiesterase-like domain (GDPD_like_SMaseD_PLD) of spider venom sphingomyelinases D was found for sequences classified into PLD1 beta family. Using RCSB PDB resource, we found that PLD1 beta shows structure similarity with 2F9R (Crystal structure of sphingomyelinase D from *Loxosceles laeta* venom or 6U8Z (Crystal Structure of Catalytic Domain of Human Phospholipase D1), and PLD2 alpha shows structure similarity with 3K72 (Structure of integrin alphaX). In general, based on sequence similarity and conserved segments of protein or nucleic acid sequences analyses of PLDs from scorpions, two types of phospholipase D (PLD) discovered in *H. saulcy*i, *A. crassicauda* and *H. lepturus* scorpion species were named PLD2 alpha and PLD1 beta. Results of the BLAST searches against NCBI-CDD and PROSITE databases are presented in Fig. 2.

**Fig. 3:**
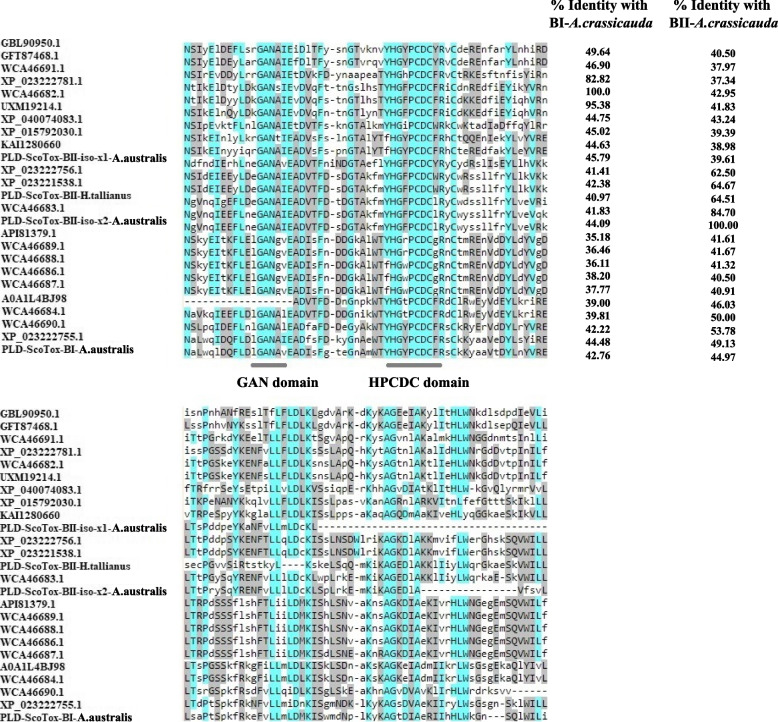
Multiple sequence alignments of the catalytic sequences of PLD1 beta in arthropods with the Clustal Omega program. Conserved amino acid residues are indicated by blue shading. All members of the PLD1 beta family share the characteristic HMGAN and HPCDC domains. Accession numbers beginning with WCA and UXM correspond to data found in this study. WCA46682.1 and WCA46683.1 represent PLD1-ScoTox-BI and PLD1-ScoTox-BII isoform × 2 from *A.crassicauda* respectively; UXM19214.1 is PLD1-ScoTox-BI isoform × 1 of *H.saulcyi*; WCA46684.1 to WCA46691.1 represent PLD1-ScoTox-BI isoforms from *H.lepturus*

The discovery of a specific diagnostic peptide pattern from the conserved sequence or known motifs of the PLD family assists in recognizing members of this highly diverged but homologous family in arthropods. To achieve this goal, the alignments of conserved segments of PLD sequences from scorpions, spiders, ticks, and mites were performed with the Clustal Omega program. Based on sequence analysis of PLD1s beta from insects and arachnids, we found that those family members had highly conserved segments (Fig. 3). All members of PLD1s beta are characterized by the sequence HMX13GANX4DX13HX2PCDC, for which two domains termed GAN (HMX13GAN) and HPCDC (HX2PCDC) can be defined for them.

Therefore, enzymes with the characteristic HMGAN and HPCDC domains are categorized as part of a PLD1 beta family. This large family was divided into two subgroups (beta I and beta II) based on sequence identity cutoff > 50% (Fig. 3) that we named Phospholipase D1 Scorpion Toxin-beta I (PLD1 ScoTox-betaI) or Dermonecrotic-like Scorpion Toxin-beta I (DScorTox-betaI), and Phospholipase D1 Scorpion Toxin-beta II (PLD1 ScoTox-betaII) or Dermonecrotic-like Scorpion Toxin-beta II (DScorTox-betaII).

Various numbers of PLD1 beta were identified from venom glands transcriptome of *H. saulcyi*, *A. crassicauda*, and *H. lepturus*, with the highest diversity observed in *H. lepturus* scorpion. In general, three PLD1 beta were discovered from venom gland of *A. crassicauda*, called PLD1 ScoTox-betaI, PLD1 ScoTox-betaII isoform × 1, and PLD1 ScoTox-betaII isoform × 2; eight from *H. lepturus*, named PLD1 ScoTox-betaI isoform × 1 to PLD1 ScoTox betaI isoform × 8; and one from *H. saulcy*i, called PLD1 ScoTox-betaI isoform × 1. The nucleic acid and protein sequence data displayed in this study are available in the NCBI under the accession number OP867052, OP867053, OP867054, OP867055, OP867056, OP867057, OP867058, OP867059, OP867060, OP867061, OP180075, and OP970823.

**Fig. 4:**
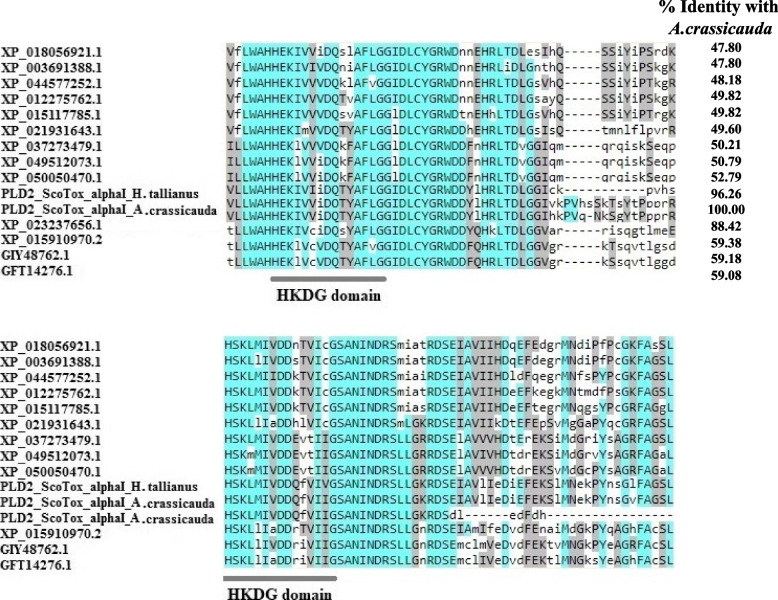
Multiple sequence alignments of the catalytic motifs of PLD2 alpha in arthropods. Conserved amino acid residues are indicated by blue shading. All members of the PLD2 alpha family share two copies of the catalytic HKDG domain

Furthermore, we aligned the PLD2 alpha sequences from scorpion species and similar sequences from other arthropods (spiders, ticks, termites, ants, flies, and wasp species) using the Clustal Omega program. We found that PLD2 alpha from arthropods is highly conserved and contains duplicate phosphodiesterase active sites. The profiles of PLD2 alpha phosphodiesterase active sites are LWAHHEKX4DQX2AFXGG and ELXYVHSKX2IXDDX3IXGSANINDRS for the first and second catalytic sequences, respectively (Fig. 4), which characterized by the sequence HxKx4Dx6G (HKDG domain). Based on these analyses of PLDs, sequences sharing two copies of the catalytic HKDG domain were categorized as part of the PLD2 alpha family. We named these PLDs, Phospholipase D2 Scorpion Toxin-alpha I (PLD2 ScoTox-alphaI). The nucleic acid and protein sequence data displayed in this study are available in the NCBI under the accession numbers OP867062 and OP820507.

### Exon–intron organization of scorpion PLDs

**Fig. 5:**
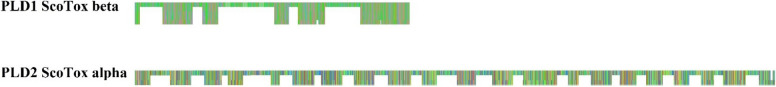
Exon–intron architecture of PLD1 beta and PLD2 alpha genes in scorpions. Colored boxes indicate exons, and the spaces between them indicate introns

**Fig. 6:**
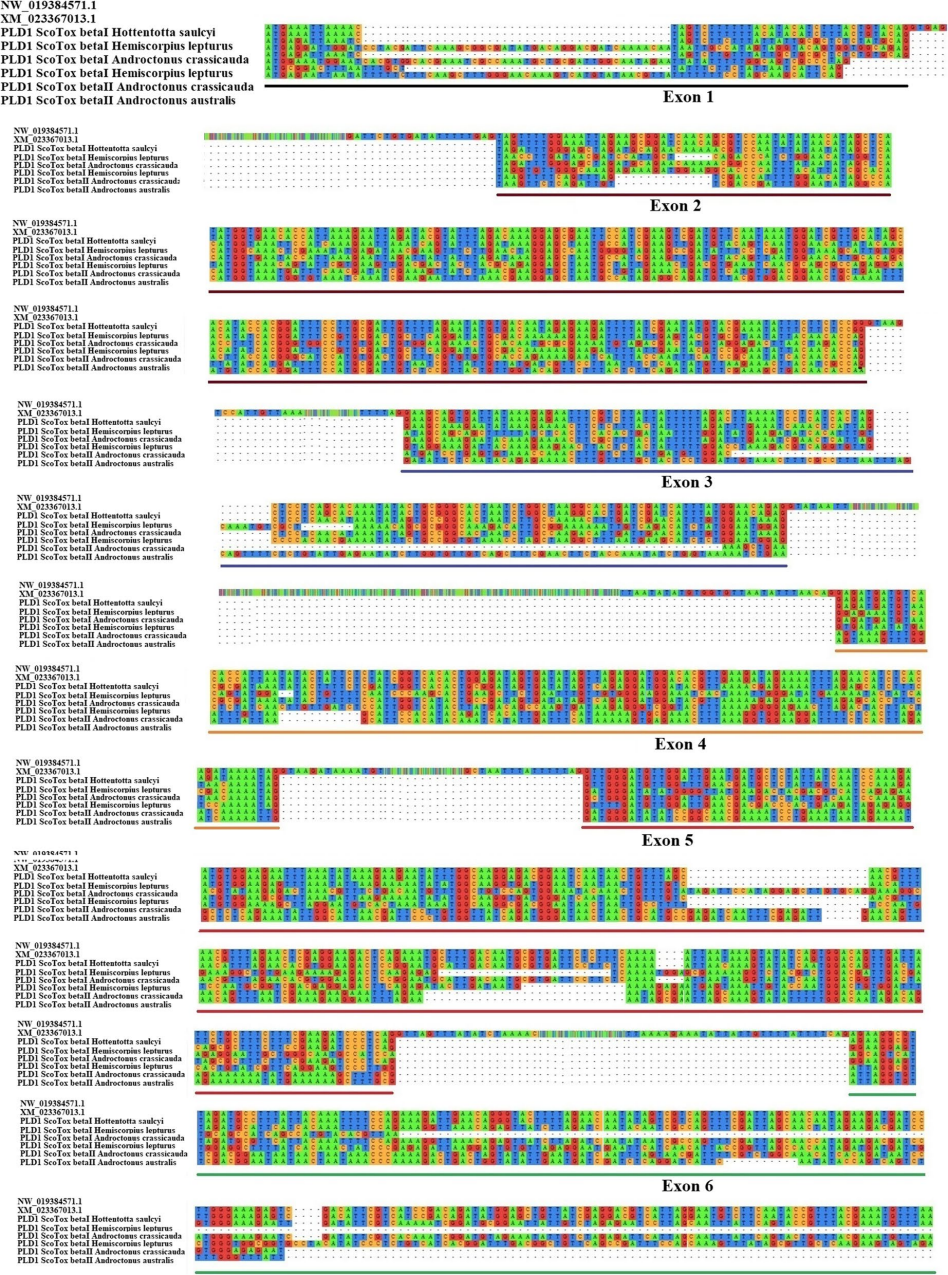
An intron–exon map of scorpion PLD1 beta genes. The genomic DNA sequence (NW_019384571.1) and mRNA sequence (XM_023367013.1) of PLDs from *C. sculpturatus* are aligned with mRNA sequences of PLD1 beta. Continuous RNA-seq reads are connected by dashed lines. The lengths of intron regions are not drawn in scale

**Fig. 7:**
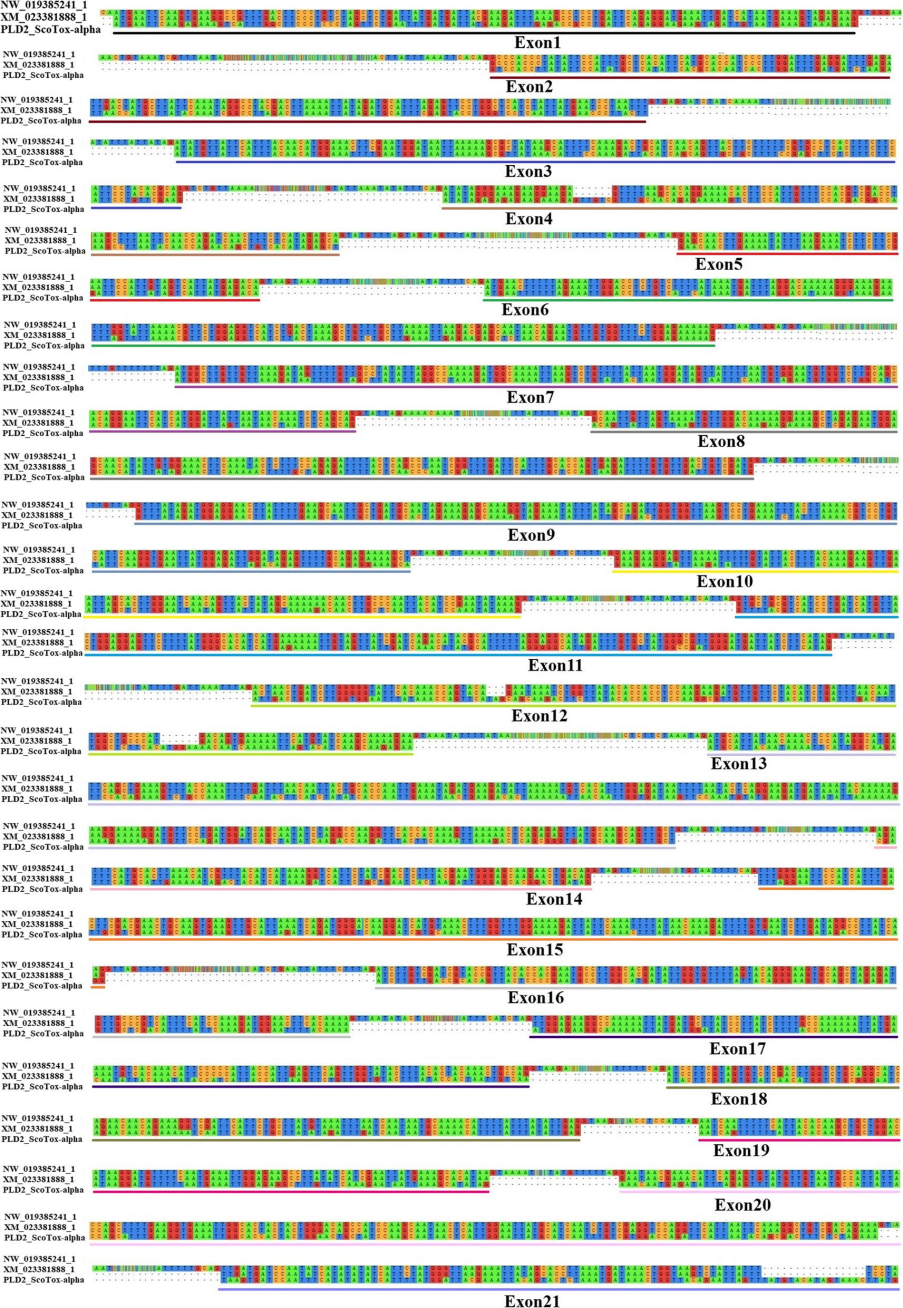
An intron–exon map of scorpion PLD2 alpha genes. The genomic DNA sequence (NW_019385241.1) and mRNA sequence (XM_023381888.1) of PLDs from *C. sculpturatus* are aligned with mRNA sequences of PLD2 alpha. Continuous RNA-seq reads are connected by dashed lines. The lengths of intron regions are not drawn in scale

The exon–intron patterns of PLD1 beta (Figs. 5 and 6) and PLD2 alpha (Figs. 5 and 7) genes were predicted by aligning the mRNA sequences of PLD1 beta or PLD2 alpha discovered in this study with the genomic sequence and mRNA of dermoncrotic toxins from *C. sculpturatus* using MAFT program. The total sequence of the PLD1 beta gene from START-codon (ATG) to STOP-codon (TAA) is around 5,400bp, and full-length cDNAs span from ~ 1,100 to 1,600bp. As shown in Fig. 6, the PLD1 beta gene was found to consist of 6 coding exons and 5 introns. Exons 1–6 vary in size from ~ 45bp (exon 1) to ~ 600bp (exon 6), whereas the introns vary in length between ~ 70bp (intron 4) to ~ 1630bp (intron 3). Exons 1 to 6 containing ~ 45, 250, 132, 125, 228, and 601bp, respectively. The introns 1 to 5 consist of ~ 1,012bp, 77bp, 1,630bp, 71bp, and 1,217bp, respectively. We found that exon 6, with a size of ~ 600bp, and intron 3, with a size of ~ 1,630bp, are the most extended regions in the PLD1 beta genes. All introns of the PLD1 beta gene follow the standard form of GT-AG.

As shown in Fig. 7, the PLD2 alpha gene is quite large and predicted to consist of 21 coding exons ranging from 68bp (exon 5) to 245bp (exon 13). The total sequence of PLD2 alpha gene and full-length cDNAs from START-codon (ATG) to STOP-codon (TAA) are ~ 45,596bp and ~ 2,800bp respectively, in which exons 1 to 21 containing ~ 120, 154, 125, 110, 68, 160, 153, 150, 166, 110, 139, 149, 245, 80, 150, 120, 123, 113, 90, 160, and 115bp, respectively. We found that the PLD2 alpha gene contains 20 GT-AG type introns. The introns 1 to 20 consist of 10,700bp, 4,102bp, 109bp, 3,384bp, 3,332bp, 5,000bp, 80bp, 8,066bp, 74bp, 168bp, 110bp, 4,446bp, 550bp, 89bp, 2,242bp, 70bp, 60bp, 84bp, 70bp, and 92bp. Exon 13, with a size of 245bp, and intron 1, with a size of 10,700bp, are the most extended regions in the PLD2 alpha genes.

### Protein structures of PLDs

Computational models of protein structures for mature PLDs were determined with I-TASSER, Phyre2, and SWISS-MODEL web servers. The quality of each model was assessed by an overall quality factor in the SAVES server and the Z-score in the ProSA-web server (Table 1). All Phyre2 generated models had less quality, according to the SAVES server assessment. Models predicted by the Swiss model with a high overall quality factor and a Z-score in the range of native conformations have the best quality. The Ramachandran map analysis of three-dimensional structures predicted by the Swiss model showed that the percentage distribution of dihedral angles of the PLD protein residues at the allowable zone is over 95%, which indicates high stability of structures; therefore, they were utilized for structural homology comparison.

**Table 1:** Quality assessment of predicted models of PLDs beta from different servers

		PHYRE2		I-TASSER		Swiss-Prot	
		o.q.f	Z-score	o.q.f	Z-score	o.q.f	Z-score
*H. lepturus*	PLD1 ScoTox-betaI isoform × 1	75.8364	-7.39	96.4413	-8.16	94.052	-7.7
	PLD1 ScoTox-betaI isoform × 3	81.5451	-6.84	95.7854	-7.18	95.6897	-7.39
	PLD1 ScoTox-betaI isoform × 4	72.9614	-7.07	85.8238	-7.34	93.9655	-7.38
	PLD1 ScoTox-betaI isoform × 5	70.9559	-6.04	90.2778	-7.78	94.4649	-7.65
	PLD1 ScoTox-betaI isoform × 6	85.6618	-6.83	89.9306	-7.77	95.9559	-7.92
*A.crassicauda*	PLD1 ScoTox-betaI	78.4387	-6.25	82.906	-4.76	94.7955	-6.49
	PLD1 ScoTox-betaII isoform × 2	66.2879	-7.11	74.6795	-8.26	93.9394	-7.43
	PLD2 ScoTox-alphaI	64.6362	-8.21	67.3052	-5.33	80.3191	-7.94
*H. saulcyi*	PLD1 ScoTox-betaI isoform × 1	70.2602	-5.67	82.8804	-5.99	94.4238	-6.79
	PLD2 ScoTox-alphaI	66.9477	-8.28	68.1023	-8.68	76.8617	-8.69

The overall quality factors (o.q.f) were estimated by SAVES server and Z-scores were estimated by ProSA-web server

### Structural homology analysis of the PLD sequences

**Fig. 8:**
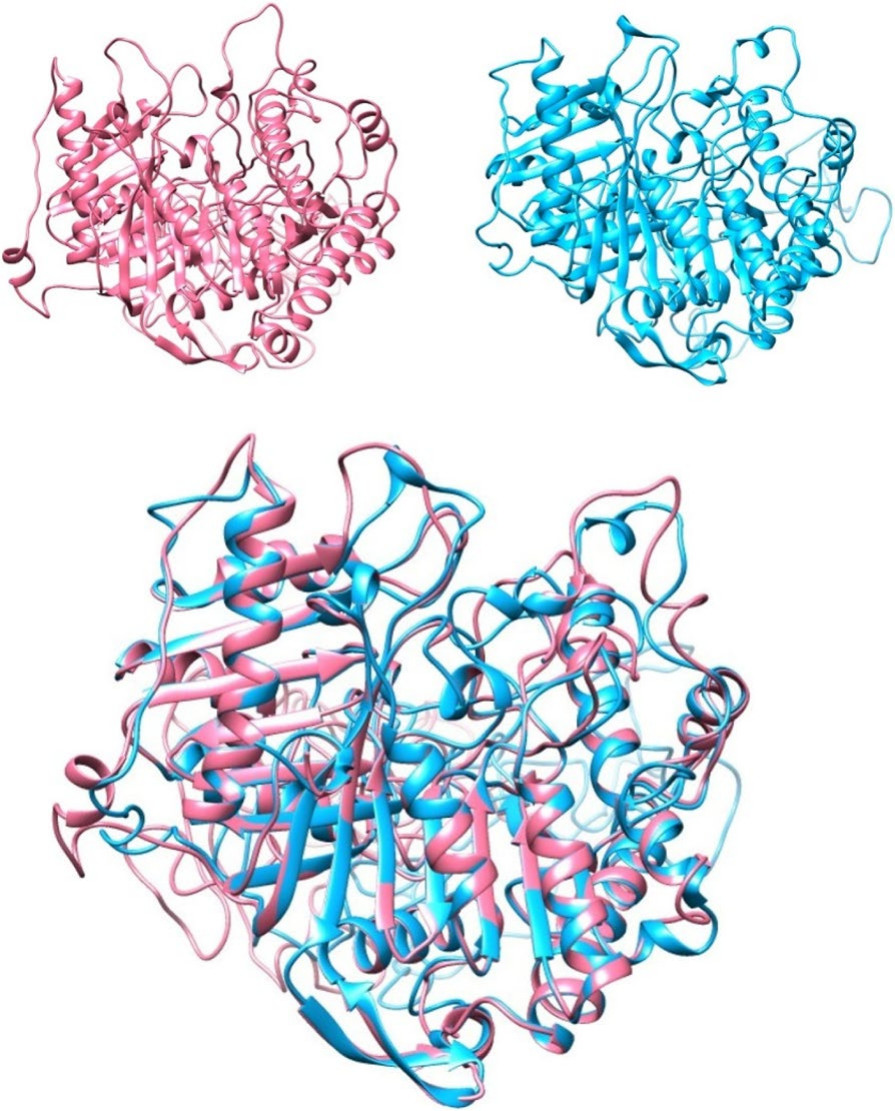
Structural modeling of PLD2s alpha from *A. crassicauda* and *H. saulcy*i by UCSF Chimera. The PLD2 alpha structure of *A. crassicauda* (top left), the PLD2 alpha structure of *H. saulcy*i (top right), and the resulting comparative model of two structures (bottom) in UCSF Chimera. In comparative models, molecular structure colors are the PLD2 alpha of *A. crassicauda* in pink and the PLD2 alpha structure of *H. saulcy*i in sky blue

**Fig. 9:**
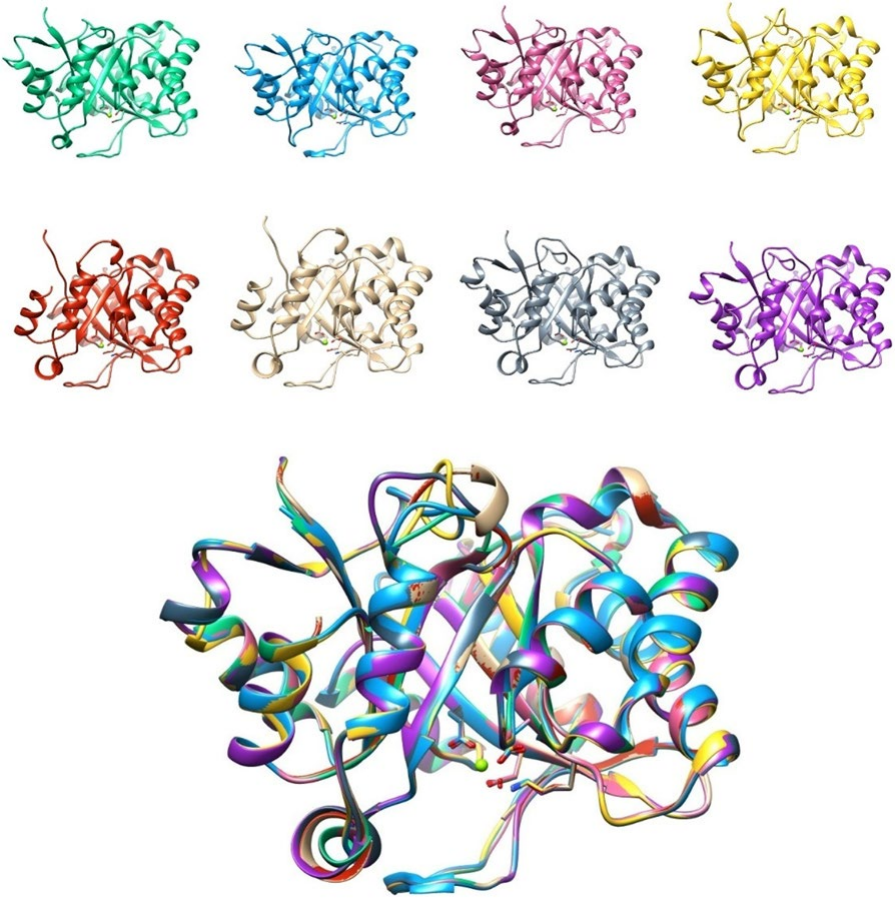
Structural modeling of PLD1 betaI and betaII from *A. crassicauda*, *H. lepturus*, and *H. saulcy*i by UCSF Chimera. The PLD1 betaI structure of *A. crassicauda* (green), PLD1 betaII structure of *A. crassicauda* (sky blue), the PLD1 betaI structure of *H. saulcy*i (pink), the PLD1 betaI structure of *H. lepturus* isoform X1 (yellow), the PLD1 betaI structure of *H. lepturus* isoform X3 (red), the PLD1 betaI structure of *H. lepturus* isoform X4 (tan), the PLD1 betaI structure of *H. lepturus* isoform X5 (slate gray), the PLD1 betaI structure of *H. lepturus* isoform X6 (purple) and the resulting eight comparative models (bottom) in Chimera. In comparative models, molecular structure colors are mentioned above in the parentheses in front of the name of each structure

The best computational models of mature PLDs were used to compare the structural homology between *A. crassicauda*–PLDs, *H. saulcy*i-PLDs, and the structure of mature dermonecrotic toxins derived from *H. lepturus*. For structural alignment and visualization, we used the molecular graphics program UCSF Chimera (ver. 1.11.2, University of California, San Francisco, CA, USA). The analysis indicated that the predicted PLD structures contain both α-helices and β-pleated sheets with significant structural homology when compared together (Figs. 8 and 9).

#### Comparative modeling analysis of PLD2s alpha

The structural comparison between the PLD2 alpha from *A. crassicauda* and the PLD2 alpha from *H. saulcyi* (Fig. 8) revealed an RMSD of 0.486 Å between all pruned atom pairs (calculated via UCSF Chimera Matchmaker), indicating that the conformations of proteins (α-helices) remained almost the same.

#### Comparative modeling analysis of PLD1s beta

Structures of PLD1 beta from *A. crassicauda*, *H. saulcyi*, and *H. lepturus* were also generally similar, and minor differences observed between PLD1 beta structures are related to the amino acid composition of the proteins (Fig. 9). The structural alignment between the modeled structure of PLD1 ScoTox-betaI from *A. crassicauda* as a template with a homology model of *A. crassicauda* PLD1 ScoTox-betaII showed an RMSD of 0.2 angstroms between 265 pruned atom pairs; (across all 272 pairs: 0.576), with a homology model of *H. saulcy*i PLD1 ScoTox-betaI had an RMSD of 0.016 angstroms between 277 pruned atom pairs; (across all 277 pairs: 0.016), and with structures of *H. lepturus* PLD1 ScoTox-betaI isoforms reported an RMSD of less than 0.2 Å on average between pruned atom pairs; (across all pairs: 0.4—0.8), which show a high degree of structural similarity between these structures.

#### Comparative modeling analysis of PLD1 ScoTox-beta with Loxosceles dermonecrotic toxins

**Fig. 10:**
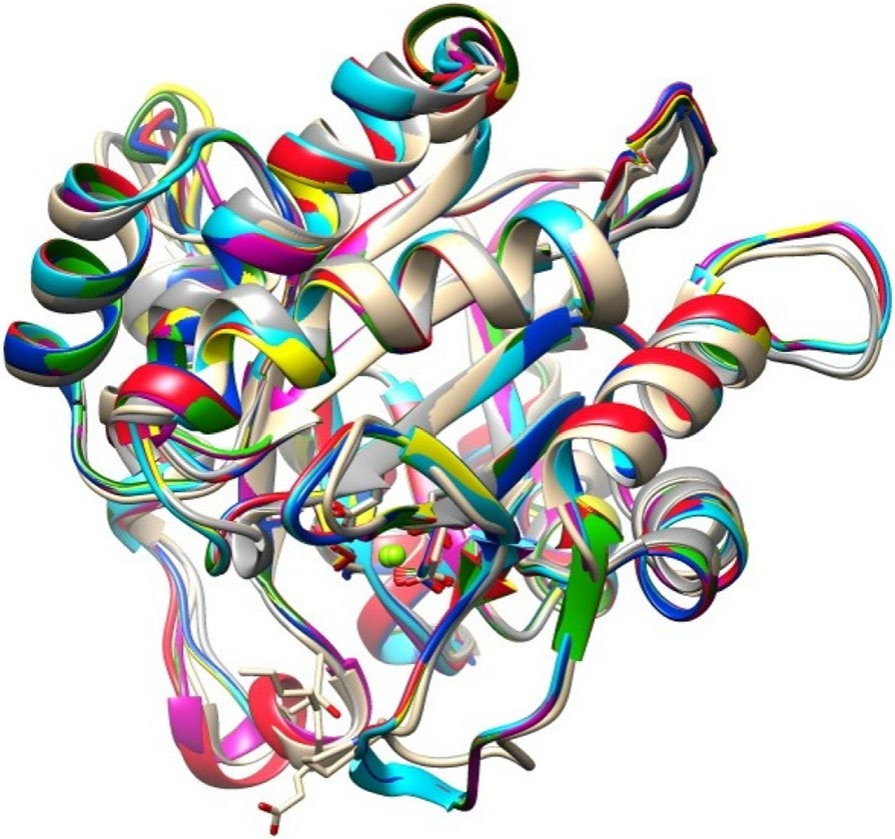
Structural alignment of the 3D structure of modeled PLD1 beta with the crystal structure of *Loxosceles* dermonecrotic toxins. The PLD1 betaI structure of *A. crassicauda* (Lime green), PLD1 betaII structure of *A. crassicauda* (sky blue), the PLD1 betaI structure of *H. saulcy*i (pink), the PLD1 betaI structure of *H. lepturus* isoform X1 (yellow), the PLD1 betaI structure of *H. lepturus* isoform X5 (red), the PLD1 betaI structure of *H. lepturus* isoform X8 (tan), the LiSicTox-alphaIA1bi (P0CE81.1) structure of *L. intermedia* (slate gray), the LiSicTox-betaIA1i (Q2XQ09.1) structure of *L. intermedia* (blue) and the PLD synthetic construct (AQZ26451.1) structure of *H. lepturus* (Olive green). In comparative models, molecular structure colors are mentioned above in the parentheses in front of the name of each structure

For in silico analysis of structural similarities and variation between predicted conformations of PLD1s beta and the crystal structure of dermonecrotic toxins, the forms of PLD1 ScoTox-beta from *A. crassicauda*, *H. lepturus*, and *H. saulcy*i were superimposed on the crystal structure of the previously determined dermonecrotic toxins from *L. intermedia* (isoforms LiSicTox-alphaIA1bi and LiSicTox-betaIA1i) and *H. lepturus* (AQZ26451.1). Significant structural homology and similarities have been found between the modeled PLDs beta and the crystal structure of dermonecrotic toxins (Fig. 10).

For detail, the RMSD between 270 out of the 275 pruned atom pairs of the LiSicTox-betaIA1i as a template with PLD1 betaI model from *A. crassicauda* is 0.17 angstroms; the RMSD between 265 out of the 273 pruned atom pairs of the template with PLD1 betaII model from *A. crassicauda* is 0.15 angstroms; the RMSD between 270 out of the 275 pruned atom pairs of the template with PLD1 betaI model from *H. saulcy*i is 0.17 angstroms; the RMSD between pruned atom pairs across all pairs of the template with PLD1 betaI models from *H. lepturus* isoforms is 0.18 angstroms on average; the RMSD between 271 out of the 275 pruned atom pairs of the template with PLD synthetic construct structure of *H. lepturus* is 0.13 angstroms; meaning that the distance between pairwise aligned backbone carbon-alpha atoms of the template and homology models was less than 0.18 angstroms, and this small RMSD indicates high structural similarity between structures (Fig. 10). In contrast, the RMSD between 256 out of the 276 pruned atom pairs of the template with LiSicTox-alphaIA1bi from *L. intermedia* is 0.8 angstroms, which is higher than the value calculated from the comparison of the structures found in this study with the template. This finding suggests that the scorpion PLD1s beta are more similar to LiSicTox-betaIA1i than to LiSicTox-alphaIA1bi.

#### Comparative modeling analysis of PLD2 ScoTox-alpha and PLD1 ScoTox-beta with Loxosceles crystal structure of sphingomyelinase D

**Fig. 11:**
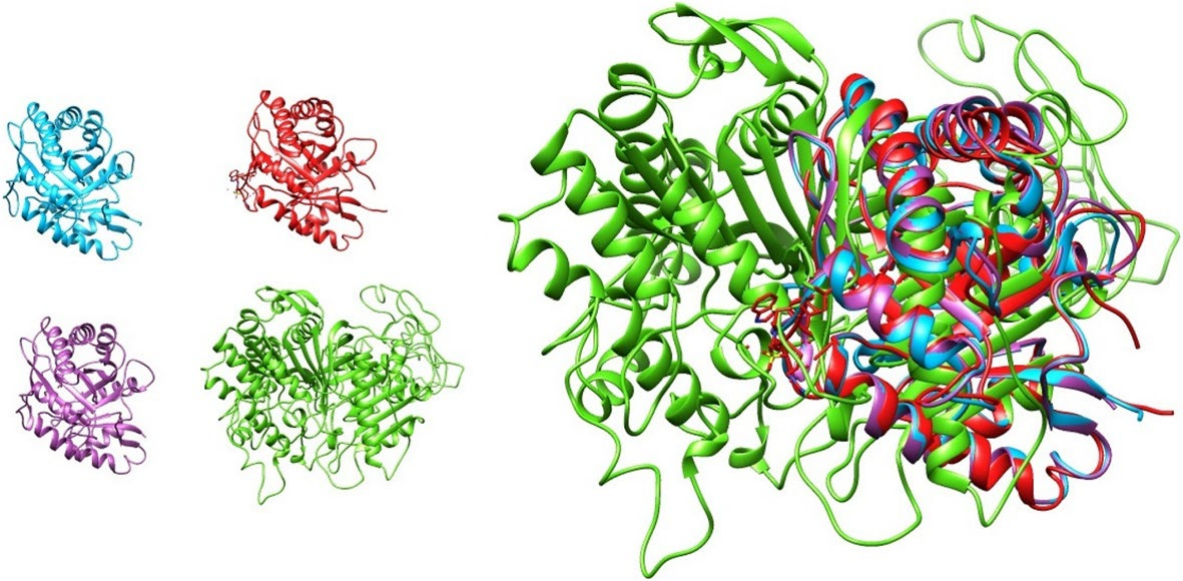
Structural modeling of the crystal structure of sphingomyelinase D from *L. laeta* venom (2f9r.pdb2), PLD1 betaI and betaII from *A. crassicauda,* and the PLD2 alpha structure of *A. crassicauda* by UCSF Chimera. The PLD1 betaI structure of *A. crassicauda* (blue), the PLD1 betaII structure of *A. crassicauda* (purple), the PLD2 alpha structure of *A. crassicauda* (green), and the crystal structure of sphingomyelinase D from *L. laeta* venom (2f9r.pdb2, red)

Two goals are followed in this analysis; first, to compare the 3D structures of PLD2 alpha with PLD1 beta of scorpions. Second, to compare the crystal structure of sphingomyelinase D from *L. laeta* as a template with the structures of PLD1 ScoTox-beta and PLD2 ScoTox-alpha. For structural comparing between the 3D structures of PLD2 alpha with PLD1 beta, the modeled structure of PLD2 ScoTox-alpha from *A. crassicauda* was superimposed on the structures of the PLD1 ScoTox-beta from *A. crassicauda* (Fig. 11). The structural alignment between the modeled structure of PLD2 ScoTox-alpha from *A. crassicauda* as a template with the model of PLD1 ScoTox-betaI showed an RMSD of 1.49 angstroms between 10 pruned atom pairs; (across all 132 pairs: 15.67), with the structure of PLD1 ScoTox-betaII had an RMSD of 1.244 angstroms between 11 pruned atom pairs; (across all 107 pairs: 14.21), which indicates deviation of atomic positions between two structures.

Furthermore, using UCSF Chimera software, we performed a structural alignment of the predicted structures of PLD2 ScoTox-alpha, PLD1 ScoTox-betaI, and PLD1 ScoTox-betaII from *A. crassicauda* with crystal structure of sphingomyelinase D from *L. laeta* venom (2f9r.pdb2, Fig. 11). Here, the aim was to compare the calculated RMSD between the backbone atoms of the sphingomyelinase D as template with PLD1 ScoTox-beta and PLD2 ScoTox-alpha. By superimposing the crystal structure of sphingomyelinase D as a template with PLD2 ScoTox-alpha, the results showed that 13 out of the 116 pruned atom pairs reported an RMSD of 1.346 Å, which indicates deviation of atomic positions in these two structures. In contrast, the RMSD between 239 out of the 275 pruned atom pairs of the template with the PLD1 betaI model, and between 241 out of the 272 pruned atom pairs of the template with the PLD1 betaII model from *A. crassicauda* are 0.88 angstroms, which indicate similar atomic positions in these structures. These findings suggest that the sphingomelinase D is more similar to beta than to alpha scorpion PLDs.

### Characterization of the gene and protein of PLD1 beta and PLD2 alpha

SignalP (version 6.0) was implemented to propose the presence, cleavage site positions, and general structure of the signal peptides. The signal peptide score, as the confidence in the signal peptide prediction, was predicted over 0.97 for all PLD1 beta members (Table 2). The general structure of the signal peptides has been suggested, and it consists of three main parts, including the N-terminal region (N-region), the intermediate hydrophobic region (H-region), and the C-terminal region (C-region). This study revealed differences in the amino acid composition and length of these three regions between PLD1 ScoTox-betaI proteins of the Buthidae and Hemiscorpiidae families, such that the longer amino acid signal peptides (32-amino acids signal peptide) were predicted for isoforms X3-X6 of *H. lepturus*.

**Table 2:** Signal peptides and their cleavage sites of PLD1-beta genes

		Signal Peptide (Sec/SPI)	Cleavage site	N-region	H-region	C-region
*H. lepturus*	PLD1 ScoTox-betaI isoform × 1	0.9991	20 and 21	2	13	5
	PLD1 ScoTox-betaI isoform × 3	0.97	32 and 33	16	13	3
	PLD1 ScoTox-betaI isoform × 4	0.9989	32 and 33	16	13	3
	PLD1 ScoTox-betaI isoform × 5	0.9951	32 and 33	16	13	3
	PLD1 ScoTox-betaI isoform × 6	0.97	32 and 33	16	13	3
*A.crassicauda*	PLD1 ScoTox-betaI	0.9991	21 and 22	2	15	4
	PLD1 ScoTox-betaII isoform × 2	0.9991	19 and 20	3	13	3
*H. saulcyi*	PLD1 ScoTox-betaI isoform × 1	0.9992	21 and 22	2	15	4

Sec/SPI: "standard" secretory signal peptides transported by the Sec translocon and cleaved by Signal Peptidase I (Lep)

In addition, such differences were also observed between the PLD1 betaI and PLD1 betaII groups of proteins. The N-regions of PLD1 ScoTox-betaI and betaII from the Buthidae family (*A. crassicauda* and *H. saulcyi*) were predicted to be composed of 2–3 amino acid residues, and they have a cleavage site at position 21 and 19, respectively. We found that the cleavage site is absent from PLD2 ScoTox-alpha proteins.

The physicochemical properties were analyzed for all mature PLDs using Expasy’s ProtParam software (Tables 3 and 4). We found that the PLD1 beta gene from scorpions encodes a mature protein with molecular weight ranging from ~ 31 to 44 kDa. Based on IP results, scorpion PLD1 beta is an acidic peptide with isoelectric points ranging from 5.5 to 6.9. The PLD1 betaI sequences in this study indicated high stability, high aliphatic indices, and negative values of GRAVY. The aliphatic indices for those protein sequences ranged from 74.74 to 83.59. Mature PLD2-alpha, with a molecular weight of approximately 128 kDa and polar nature (negative GRAVY value), has a basic isoelectric point (~ 9), and high aliphatic index (Table 4).

**Table 3:** Physiochemical properties of PLD1-beta genes

		Mature protein amino acids No	Molecular weight	Theoretical pI	Instability index	Aliphatic index	GRAVY
*H. lepturus*	PLD1 ScoTox-betaI isoform × 1	289	33,138.65	6.91	36.46	83.59	-0.497
	PLD1 ScoTox-betaI isoform × 3	270	31,106.86	5.82	37.61	75.81	-0.569
	PLD1 ScoTox-betaI isoform × 4	270	31,183.90	5.63	37.61	74.74	-0.612
	PLD1 ScoTox-betaI isoform × 5	296	34,224.60	5.81	28.78	81.01	-0.537
	PLD1 ScoTox-betaI isoform × 6	296	34,111.44	5.81	28.10	81.01	-0.550
*A.crassicauda*	PLD1 ScoTox-betaI	378	43,823.41	6.29	38.19	82.30	-0.494
	PLD1 ScoTox-betaII isoform × 2	320	37,498.90	5.55	41.66	94.75	-0.350
*H. saulcyi*	PLD1 ScoTox-betaI isoform × 1	378	43,689.21	6.41	32.41	83.07	-0.499

*MW* Molecular weight, *GRAVY* Grand average of hydropathicity

**Table 4:** Physiochemical properties of PLD2 ScoTox-alpha genes

	Molecular weight	Theoretical pI	Instability index	Aliphatic index	GRAVY	Signal Peptide (Sec/SPI)
*A. crassicauda*	128307.65	9.07	44.89	91.88	-0.348	0
*H. saulcyi*	128165.45	9.10	44.38	91.27	-0.347	0

### Phylogenetic relationships of the arthropods

**Fig. 12:**
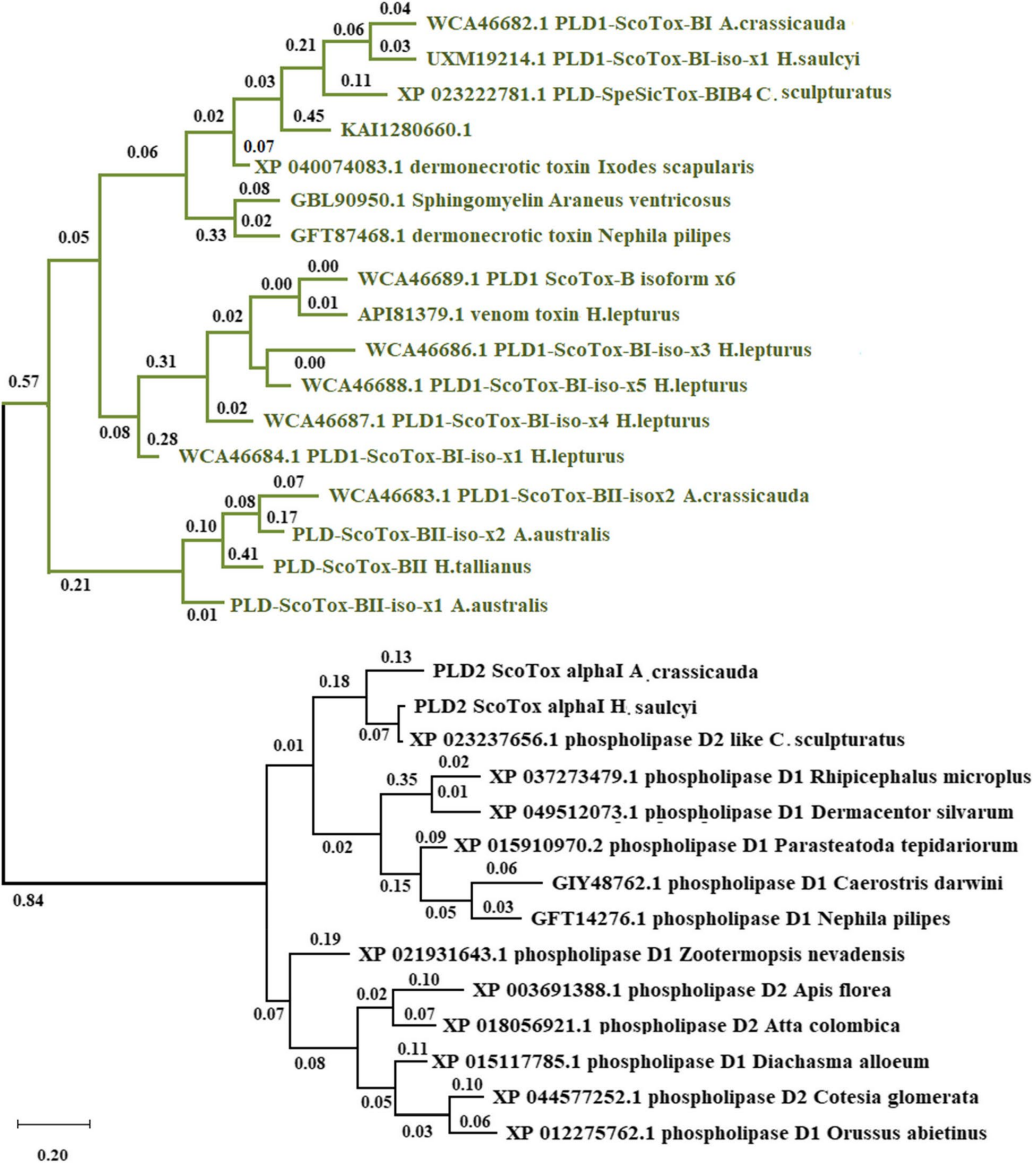
Phylogenetic analysis of the arthropods PLD family. A phylogenetic tree of scorpions, spiders, ticks, termites, ants, flies, and wasp species PLDs, constructed by the maximum likelihood algorithm using the PhyML 3.0 program (see Materials and Methods). PLD2 alpha and beta subfamilies are indicated by black and green color, respectively. Phylogenetic tree with branch length showing relationships of scorpions PLD genes to other PLD genes of known sequence

Phylogenetic analyses of the PLD proteins were conducted using the PhyML 3.0 program ^10^ to evaluate the evolutionary relationship of PLD proteins in arthropods. To build the phylogenetic tree, we included dermoncrotic toxins sequences from this study, alongside available PLD homologous peptides in GenBank from *A. australis* and *H. trilineatus* scorpions, and six out-groups, including spiders, ticks, termites, ants, flies, and wasp species (Additional file 1). Our phylogenetic analysis classified the PLD family of proteins into two main subfamilies, PLD1 beta and PLD2-alpha (Fig. 12). Clade 1 consists of PLD1 beta and clade 2 includes PLD2 alpha, which clade 1 is further divided into the subclades betaI and betaII. The phylogenetic tree represents that PLD2 alpha members share a close evolutionary relationship, as well as PLD1 beta members of different arthropod species. However, there are long evolutionary distances between PLD2 alpha and PLD1 beta.

The analysis of PLDs showed that *A. crassicauda* and *H. saulcy*i PLD members have a close phylogenetic relationship with their other Buthidae family orthologs. However, they have a long phylogenetic distance to their *H. lepturus* orthologs. Furthermore, among all arthropods, the closest phylogenetic relationship of PLD members was observed between scorpions and spiders.

## Discussion

Lethal potency, neurotoxicity, and cytotoxicity of venom of different scorpion species have been reported in human [26] mice [27] and *Sesamia nonagrioides* [28]. In terms of medical importance, the venoms of *A. crassicauda, H. saulcyi,* and *H. lepturus* play an essential role in the envenoming of humans [2]. For this purpose, transcriptomic analyses on the venom glands of these three scorpion species from two families of Buthidae (*A. crassicauda and H. saulcyi*) and Hemiscorpiidae (*H. lepturus*) were conducted in order to identify enzymes that potentially play essential roles during scorpion envenoming. In the last two decades, numerous studies have evaluated various components in the venom of poisonous animals, including scorpions, using genetic engineering strategies, transcriptomics, and proteomics approaches [13, 15, 29, 30], some of which have identified toxin transcript sequences similar to phospholipase D responsible for neurotoxicity and lethality [7, 17–19, 31]. Phospholipase D, a superfamily of transphosphatidylase, generates phosphatidic acid as a lipid second messenger through hydrolysis of the phosphatidylcholine (PC) [32]. Previous research have shown that PLD plays its role in several cellular processes, including hypertension, vesicular trafficking, exocytosis, endocytosis, phagocytosis, regulation of cellular metabolism, and tumorigenesis, indirectly through its catalytic products, phosphatidic acid [32–34]. In Vitro study of recombinant dermonecrotic toxins from brown spider (*L. intermedia*) and scorpion of *H. lepturus* venom showed that those venom components caused a wide range of pathophysiological effects in rabbit and mice, including inducing a local inflammatory response, dermonecrosis, lysis of red blood cells, platelet adhesion, increased permeability of blood vessels and acute kidney injury [16, 29, 35]. These findings made us focus on the identification and investigation of scorpion venom PLDs.

### Identification and classification of the PLD isoforms

The current study was conducted to report transcriptome analysis of venom glands of *H. saulcyi*, *H. lepturus*, and *A. crassicauda* with insight into the identification, classification, and characterization of the putative phospholipase D. This report marks the first evidence of PLDs in the venom of *H. saulcyi* and *A. crassicauda* scorpions. Multiple transcripts with high sequence similarity to PLDs were identified in the venom glands transcriptome of three studied scorpions by BLAST searching against the local PLD database. This study sequence similarity analysis showed that two forms of phospholipase D are encoded by the scorpion’s venom gland.

First, recombinant PLD of venom, with a molecular weight of 32–35 kDa, were purified from the venom of the spider [36], then a 33 kDa protein representing sphingomyelinase D and dermonecrosis activity was purified from the venom of *H. lepturus* [16]. Although after reporting the first evidence of phospholipase D activity in a scorpion venom of *H. lepturus* [16], several studies, including the Reference Sequence (RefSeq) Genome Assembly bioproject of *C. sculpturatus* (PRJNA422877), venom gland transcriptomic analyses of *H. lepturus* [17], *M. gertschi* (JAW07090.1) [7] and *P. schwenkmeyeri* [7] identified toxin transcript sequences similar to phospholipase D as well as, recombinant phospholipase D1 from *H. lepturus* [19, 31], so far characterization of catalytic motifs of scorpions PLDs and classification of them have not been done. Given the fact that conserved protein sequence regions are beneficial for identifying, grouping, and studying protein families due to their importance in terms of the structure or function of the molecule [37], we used motif extraction to classify the newly found sequences. Interestingly, it can be inferred from our motifs and domains classification that venom gland of scorpions encodes two PLD isoforms; PLD1 beta, known as Phospholipase D1 Scorpion Toxin-beta (PLD1 ScoTox-beta) or Dermonecrotic-like Scorpion Toxin-beta (DScorTox-beta), and PLD2 alpha that known as Phospholipase D2 Scorpion Toxin-alpha I (PLD2 ScoTox-alpha I) or Dermonecrotic-like Scorpion Toxin-alpha I (DScorTox-alpha I). Indeed, we found that PLD1 ScoTox-beta and PLD2 ScoTox-alpha differ in their regulatory motifs. By multiple sequence alignments of the catalytic motifs of PLD superfamily from arthropods, it was found that the PLD1 beta group lacks the HKD conserved motif. In contrast, the PLD2 alpha group has duplicate HKD motifs and new key residues were identified as common features of PLD2 alpha (HxKx4Dx6G) and PLD1 beta (GAN and HPCDC).

Consistent with our study, two PLD isoforms (PLD1 and PLD2) have already been found in mammals [38], plants, and yeast [39]. In another classification, the PLD superfamily from plants, microbes, and eukaryotes was classified into two groups, HKD PLD and non-HKD PLD, based on the presence of the HKD catalytic motif [40].

In addition, based on sequence identity cutoff > 50%, two classes of the PLD1 beta gene; betaI and betaII, were identified from the venom glands transcriptome of *H. saulcyi*, *A. crassicauda*, and, *H. lepturus* (Fig. 3). These results were further investigated by structural homology analysis (Fig. 9), which uses the RMSD for quantitative measurement of similarity between two or more protein structures. It was reported that the smaller the RMSD between two structures, the more similar are these two structures [41]. Accordingly, PLDs with the smallest RMSD between structures were grouped into one category. In general, the results of the structural homology analysis were consistent with the sequence similarity analysis. Interesting, we found that homologous PLDs retain highly similar 3D structures even at lower sequence identity. The conservation of the three-dimensional structures of proteins during molecular evolution may be the cause of this difference; and on the other hand, it may indicate the functional conservation of this gene family. Similar to the results of the conserved regions, in our phylogenetic analysis of PLDs, PLD1 beta, and PLD2 alpha clustered as a unique subfamily, while PLD1 betaI and betaII were grouped in one cluster; these results are also consistent with the results of sequence alignment.

Interestingly, the results of our motif extraction, sequence and, structural homology searches revealed that scorpion PLD1s beta share homology with secreted mammalian glycosylphosphatidylinositol phospholipase D (GPI-PLD), phosphatidylcholine-specific PLD (PC-PLD), and *Loxosceles* PLDs (*L. intermedia* and *L. laeta*). Meanwhile, PLD2s alpha share a common feature with PX/PH proteins.

Consistent with our study, the GPI-PLD, PC-PLD, and *Loxosceles* PLDs, which share distant homologies, are grouped as non-HKD enzymes, and plant and mammalian PXPH-PLD enzymes are grouped as HKD PLDs [33, 40, 42]. Citing that *Loxosceles* PLD with SMase activity had been classified as lacking HKD enzymes [42], and considering previous research in which comparing the recombinant phospholipase D toxins from *Hemiscorpius* and *Loxosceles* venom, the equal ability of these two PLDs for hydrolyzing lysophosphatidylcholine (LPC) and sphingomyelin was shown [43], so the scorpion PLD1 beta enzymes can be considered structurally and functionally homologous with most of the PLDs from *Loxosceles* (this must be proven experimentally), while scorpion PLD2 alpha is estimated to have similar functions to several members of mammalian PLDs that bear a PX domain and are represented by a duplicated HKD motif. It has been explained that these members of mammalian PLDs, in addition to lipase activity, hydrolyze phosphodiester bonds through the HKD catalytic motif [42]. Since previous studies have reported different activities for these three PLDs, GPI-PLD hydrolyzes GPIs to produce PA and a free head group, PC-PLD catalyzes the hydrolysis of PC to produce phosphatidic acid and choline, and *Loxosceles* PLDs exhibit SMase activity [33, 40, 42]; thus, more accurate classification of PLDs in scorpions requires studying the physicochemical properties of these isoforms in scorpions.

### Characterization of the gene and protein of PLD1 beta and PLD2 alpha

We found that PLD1 beta, with molecular weights ranging from 31 to 43 kDa, is an acidic protein containing a signal peptide, and PLD2 alpha, with molecular weights of 128 kDa, is a basic protein without a known signal peptide. Intron–exon pattern analysis of PLD1 beta and PLD2 alpha genes resulted in the prediction of 6 and 21 exons, respectively. The different characteristics of PLD1 beta and PLD2 alpha prove their difference in localization or functions. Basically, phospholipases differ in structure, function, regulation, and mode of action, which are divided into two general groups, the acylhydrolases and the phosphodiesterases [44]. Functionally, most cells comprise a large number of phospholipases that can be acted as secreted forms, membrane associated, and intracellular located [44]. Overall, based on the results of this study and previous studies, the difference between PLD1 and PLD2, in addition to the primary sequence and function, is also in their intracellular location and the types of activating proteins [39, 42]. Initial studies based on overexpression and monoclonal antibody studies suggested that PLD1 is primarily found in intracellular membranes, while PLD2 is located on the plasma membrane [10, 39, 45]. These studies, based on the localization and activating proteins of PLDs, support the role of both PLDs in facilitating receptor-mediated endocytosis, where PLD1 mediates vesicular traffic, which is part of the main activities of PLDs reported in previous studies, and PLD2 facilitates endocytosis of the angiotensin II type 1 receptor. However, the role of PLDs in endocytosis may be complex; likely, that both PLD1 and PLD2 work together to stimulate receptor endocytosis, such that the activation of one leads to the activation of the other [45]. Basically, phospholipases differ in structure, function, regulation, and mode of action, which are divided into two general groups, the acylhydrolases and the phosphodiesterases [44]. Functionally, most cells comprise a large number of phospholipases that can be acted as secreted forms, membrane associated, and intracellular located [44]. In any case, the available data about the role of each of these two are few, and more studies are needed in this matter. However, the difference between these two PLDs in the presence of the signal peptide sequence can be another reason for the difference in their cellular location. Because all proteins from eukaryotic cells are synthesized in cytoplasm and signal peptides direct link proteins to their specific locations. Here, we reported the high structural similarity between PLD1 ScoTox-beta from *A. crassicauda, H. saulcyi,* and *H. lepturus* and crystal structures of sphingomyelinase D and dermontcrotic toxins of the *Loxosceles* venom. This level of structural similarity provides new insights for the future discovery of the sphingomyelinase and dermontcrotic activities in the venom of these scorpions.

## Conclusions

Many biotechnological studies have suggested that PLDs derived from scorpion and spider venom have the potential to be used in next-generation immunization strategies due to their important structural and functional diversity. This study provides relevant findings for improving their use as valuable tools for developing antivenoms and new drugs. In this study, we reported the transcriptome information of *H. saulcyi*, *A. crassicauda*, and *H. lepturus*, emphasizing the identification and classification of PLDs, which can provide insight into their identification and classification in arthropods. In scorpions, genes encoding PLD isoforms are classified into two groups: alpha and beta. This classification is highly based on similarities in sequence identity, motifs and domain conservation, exon–intron architecture, protein structures, and phylogenetic analysis. It can serve as a reference in grouping newly discovered PLDs from scorpions.

### Supplementary Information

**Additional file 1.** Phospholipase D protein sequences are used to generate the multiple sequence alignment for the detection of a specific diagnostic peptide pattern from the conserved sequence and phylogenetic analyses.

## Data Availability

The mRNA and protein sequences reported in this paper are appearing in the NCBI under the accession numbers OP867052, OP867053, OP867054, OP867055, OP867056, OP867057, OP867058, OP867059, OP867060, OP867061, OP180075, OP867062, OP970823, and OP820507.

## Abbreviation

PLD: Phospholipase D
SM: Sphingomyelin
C1P: Ceramide 1-phosphate
WHO: World health organization
RIN: RNA Integrity Number
ORFs: Open Reading Frames
CDD: Conserved Domain Database
PLD1 ScoTox-betaI: Phospholipase D1 Scorpion Toxin-beta I
DScorTox-betaI: Dermonecrotic-like Scorpion Toxin-beta I
PLD1 ScoTox-betaII: Phospholipase D1 Scorpion Toxin-beta II
DScorTox-betaII: Dermonecrotic-like Scorpion Toxin-beta II
PLD2 ScoTox-alphaI: Phospholipase D2 Scorpion Toxin-alpha I
DScorTox-alphaI: Dermonecrotic-like Scorpion Toxin-alpha I
LPC: Lysophosphatidylcholine
LPA: Lysophosphatidic acid
PC: Phosphatidylcholine
PI: Phosphatidylinositol
GPI-PLD: Glycosylphosphatidylinositol phospholipase D
PC-PLD: Phosphatidylcholine-specific PLD
SMase: Sphingomyelinase
RMSD: Root-mean-square distance
