# Supplementary material for: Genome-wide identification, structural homology analysis, and evolutionary diversification of the phospholipase D gene family in the venom gland of three scorpion species
Source: BMC Genomics. 2023 Dec 4;24:730. doi: 10.1186/s12864-023-09851-y (PMC10694872; doi:10.1186/s12864-023-09851-y)
Supplement: Supplementary file 1 — Additional file 1. Phospholipase D protein sequences are used to generate the multiple sequence alignment for the detection of a specific diagnostic peptide pattern from the conserved sequence and phylogenetic analyses. [file 12864_2023_9851_MOESM1_ESM.docx]

**Genome-wide identification, structural homology analysis, and evolutionary diversification of the phospholipase D gene family in the venom gland of three scorpion species**

**Masoumeh Baradaran^1^ and Fatemeh Salabi^2^***

**Additional file 1.** Phospholipase D protein sequences are used to generate the multiple sequence alignment for the detection of a specific diagnostic peptide pattern from the conserved sequence and phylogenetic analyses.

| Taxon | GenBank ID |
| --- | --- |
| *Hemiscorpius lepturus* | A0A1L4BJ98.1 |
| *Hemiscorpius lepturus* | API81379.1 |
| *Centruroides sculpturatus* | XP_023221538.1 |
| *Centruroides sculpturatus* | XP_023222781.1 |
| *Centruroides sculpturatus* | XP_023222756.1 |
| *Centruroides sculpturatus* | XP_023222755.1 |
| *Centruroides sculpturatus* | XP_023222769.1 |
| *Centruroides sculpturatus* | XP_023237656.1 |
| *Megacormus gertschi* | JAW07090.1 |
| *Araneus ventricosus* | GBL90950.1 |
| *Nephila pilipes* | GFT87468.1 |
| *Ixodes scapularis* | XP_040074083.1 |
| *Tetranychus urticae* | XP_015792030.1 |
| *Halotydeus destructor* | KAI1280660.1 |
| *Parasteatoda tepidariorum* | XP_015910970.2 |
| *Caerostris darwini* | GIY48762.1 |
| *Nephila pilipes* | GFT14276.1 |
| *Rhipicephalus microplus* | XP_037273479.1 |
| *Dermacentor silvarum* | XP_049512073.1 |
| *Dermacentor andersoni* | XP_050050470.1 |
| *Zootermopsis nevadensis* | XP_021931643.1 |
| *Orussus abietinus* | XP_012275762.1 |
| *Diachasma alloeum* | XP_015117785.1 |
| *Cotesia glomerata* | XP_044577252.1 |
| *Atta colombica* | XP_018056921.1 |
| *Apis florea* | XP_003691388.1 |
